# Dissecting the Urge to Create

**DOI:** 10.1371/journal.pbio.0020047

**Published:** 2004-02-17

**Authors:** Nancy C Andreasen

## Abstract

A neuropsychiatrist reviews "The Midnight Disease: The drive to write, writer's block and the creative brain"

Creative human beings are the torch-bearers of civilization. How does their creativity arise? What causes some minds/brains to achieve awe-inspiring artistic or scientific achievements? We cannot help but be fascinated by the fact that Shakespeare—a merchant's son with “small Latin and less Greek”—could emerge from the “nowhere” of rural Stratford to create the richest literary treasure in the English language. We wonder how Michelangelo—a stonecutter's son who also came from a rural nowhere—found within himself the vision to see the shape of David in a block of discarded marble or the apolcalyptic fresco of *The Last Judgment* on the wall of the Sistine Chapel. What genetic influences shaped their brains to create—and to create these very specific wondrous things? How did their environments promote or impede them? Would Michelangelo have been great without the patronage of the Medicis or the competitive edge induced by Leonardo? Great art and great science are indeed often forged in the smithy of pain—with the fire fueled by self-doubt, obsessive preoccupation, sorrow, depression, competition, or economic needs.

*The Midnight Disease: The Drive to Write, Writer's Block, and the Creative Brain* by Alice Weaver Flaherty unites two intrinsically fascinating domains of knowledge—the workings of the brain and the nature of creativity. Its author, a neurologist who has also become a writer by virtue of having published her first nonacademic book, draws on her knowledge of neuroscience, her medical career as a clinician, and her experiences as a patient. Early in the book, she describes her own hospitalization for manic-depressive illness, a disclosure that implicitly places her in the pantheon of other artists who have suffered from serious mental illness and provides her with lustre-by-association. The result of all these juxtapositions is, however, a somewhat disconcerting blend of pop-science and pop-confessional genres. The author frequently talks to us in the first person, but one is not quite sure which person (the neuroscientist, the doctor, or the patient) is actually speaking. In other words, this book has a jarring lack of a strong single voice, despite a knack for often finding a fine turn-of-phrase or a clever word choice.

Given that the book topic is promising and that the author can often write very well, it is dismaying that this book is not better than it is. It is written for the intelligent lay public, many of whom avidly collect and read “brain books” to expand their minds. Most painful is the fact that this book is filled with factual errors, glib and misleading generalizations, and careless misstatements. Perhaps most shocking and most erroneous, we are told (by a neurologist!) that “The tips of the temporal lobe can be lopped off without much changing a person's behavior.” HM, the most famous patient to receive bilateral temporal lobectomy, remains frozen in a past linked to a never-changing present because he lost the capacity to retain new memories. Temporal lobe syndromes are discussed more accurately later in the book, but that is a weak excuse for this early error.

We are also told that “manic depression is a genetically transmitted syndrome” (when, in fact, no replicable genetic loci have yet been identified), that “a very high proportion of manic depressives become writers” (the lifetime prevalence rate of bipolar disorder is approximately 1%, and only a tiny proportion of that 1% are writers), and that “electrophysiology, because it is dangerous, is rarely performed” (electrophysiology tools—e.g., the study of evoked potentials or electroencephalograms—are noninvasive and frequently used; recordings of the activity of individual neurons with electrodes placed in the gray matter are indeed rare, but nothing from the context suggests that this particular type of electrophysiology is being discussed). There are many more such careless misstatements. The intelligent lay reader deserves better than this.

The book raises and addresses a variety of interesting questions that have intrigued many thoughtful people for more than two millennia. What is the nature of creativity? What is the difference between skill and creativity? What is the relation between mental illness and creativity? Is creativity inhibited when mental illnesses are treated? What is the relation between mind and brain? The book also addresses some unique and interesting twists on these questions. Its focus is the domain of writing, drawing from the author's own experience of a compulsion to write, or hypergraphia, following a pyschic break. What is the relationship between hypergraphia and the brain? Between writer's block and the brain? Are these problems always pathological, or do they sometimes enhance creativity? Does that college student who can't finish a term paper have a “disease”? Can “mind-expanding” drugs that affect the brain enhance creativity?

In short, *The Midnight Disease* raises many important questions, but fails to address them completely and accurately. There is much more to learn, and much more to say, about the nature of creativity, its origins in the mind/brain and in the human genome, and its boundaries with health and disease.

